# A replication study of schizophrenia-related rare copy number variations in a Han Southern Chinese population

**DOI:** 10.1186/s41065-016-0025-x

**Published:** 2017-01-14

**Authors:** Jianmin Yuan, Jianlin Hu, Zhiqiang Li, Fuquan Zhang, Dexiang Zhou, Chunhui Jin

**Affiliations:** 1 Wuxi Mental Health Center, Nanjing Medial University, 156 Qianrong Road, Wuxi, 214151 Jiangsu Province China; 2 Wuxi Second Traditional Chinese Medicine Hospital, Wuxi, 214151 Jiangsu Province China; 3 Bio-X Institutes, Key Laboratory for the Genetics of Developmental and Neuropsychiatric Disorders (Ministry of Education), Shanghai Jiao Tong University, Shanghai, China

**Keywords:** Schizophrenia, Copy number variants, Han Chinese

## Abstract

**Background:**

Schizophrenia (SCZ) is a common, complex and severe psychiatric disorder associated with many different genetic and environmental risk factors. Evidence from genetic studies has revealed the role of genome structural variations, specifically copy number variants (CNVs), in the etiology of SCZ. Nevertheless, the occurrence of CNVs and their relation to SCZ has remained relatively unstudied in the diverse Han Chinese population.

**Results:**

We used a case/control paradigm, including 476 cases and 1023 controls. All samples were genotyped using the Axiom® Exome Genotyping Arrays. Four CNVs, including two deletions and two duplications, were detected in this study. Notably, the 16p11.2 duplication from 29.3 Mb to 29.6 Mb was detected in four cases (0.84%) and one control (0.098%) (*p* = 0.0377).

**Conclusions:**

The results highlight the potential role of these deletions and duplications in the development of SCZ. Clearly, larger sample sized studies are needed for a careful localization of these CNVs and to possibly detect more deletions and/or duplications, associated with the development of SCZ in the Han Chinese population.

**Electronic supplementary material:**

The online version of this article (doi:10.1186/s41065-016-0025-x) contains supplementary material, which is available to authorized users.

## Background

The role of genome structural variations, specifically copy number variants (CNVs), in the development of mental disorders is a growing area of study. Due to the potential polygenic effects and the capacity to alter gene dosage, CNVs may have dramatic consequences on expression levels and therefore phenotype. Szatkiewicz et al hypothesize that CNV deletions are more severe compared to CNV duplications, due to the loss of function effect [1]. Furthermore, large CNVs are more severe than small CNVs, which affect fewer genes and regulatory regions. Because of their direct impact on gene modification, CNVs play a major role in evolution and have the potential to disturb some genomically unstable areas. For instance, the 1q21.1 region is associated with neurological development and thus could have impacted brain evolution. Therefore, deletion of 1q21.1 could explain the neurological and psychiatric conditions observed in patients carrying this CNV. The larger expression of the genome by brain tissue compared to other tissues explains the rationale for selecting CNVs as likely candidates for modulating psychiatric illnesses. As a result of the cost associated with whole genome sequencing, exome arrays have emerged as a viable option for the detection of both single nucleotide polymorphisms (SNPs) and CNVs. Only protein-coding DNA will be examined, thereby providing an approximate portrayal of the genomic expression. Exome arrays have both confirmed SNPs previously established by genome wide association studies (GWAS), as well as identified novel SNPs associated with a wide-range of diseases. Exome sequencing has been utilized for CNV analysis in the genome analysis of Swedish patients with schizophrenia (SCZ) and has confirmed some of the previously determined CNVs from GWAS [1]. Nevertheless, the diverse Han Chinese population represents a major group that has remained relatively unstudied with regards to the occurrence of CNVs and their relation to mental health disorders, such as SCZ. Several previous studies implicated numerous rare CNVs in SCZ, including the 1q21.1 deletion [2], 15q11.2 deletion [3], Williams-Beuren syndrome (WBS) region duplication [4] and 16p11.2 duplication [1]. These new findings raise fundamental clinical and scientific questions concerning classification of major neuropsychiatric disorders, modes of inheritance, diagnostics, and genetic counseling. None of these CNVs have been investigated in Chinese Han population. In this paper we analyze the exome SNP array data in 476 SCZ cases and 1023 healthy controls taken from southern China for the presence of CNVs, particularly focusing on the four CNVs listed above.

## Results and Discussion

The 16p11.2 duplication from 29.3 Mb to 29.6 Mb was detected in four cases (0.84%) and one control (0.098%) (*p* = 0.0377, Fisher’s exact test) (Table 1). A previous investigation by McCarthy et al [5] reported an association of an adjacent duplication from 29.6 Mb to 30.2 Mb within 16p11.2 with SCZ (*p* = 1.2 × 10^−5^ for the initial cohort, *p* = 0.022 in the replication cohort). A subsequent meta-analysis from a follow-up study [5] involving 8,590 individuals with schizophrenia, 2,172 with developmental delay or autism spectrum disorders (ASD), 4,822 with bipolar disorder, and 30,492 controls reported a p value of 4.8 × 10^−7^ for association with SCZ. The authors also reported an association between the 16p11.2 duplication and both bipolar disorder and autism. Alternatively, the 16p11.2 deletion has been associated with autism and developmental disorders, suggesting an overlapping etiology between SCZ, ASD and other developmental disorders. The 16p11.2 duplication is also associated with a decrease in brain size compared to 16p11.2 deletion, and it is worth noting the possible connection between autism and increased brain volumes, not only in the content of McCarthy et al, but also in other studies.

**Table 1:** The significant level of the identified rare CNVs

Locus	Position in Mb (hg19)	Cases (*N* = 476)	Control (*N* = 1023)	OR	Fisher's P	Cases from previous studies^a^ (%)	Controls from previous studies^a^ (%)	Frequency in Chinese population^b^ (%)
1q21.1 del	chr1:146.5–147.8	1	0	NA	0.3175	0.170	0.016	0.092
15q11.2 del	chr15:22.7–23.1	1	1	2.15	0.5344	0.640	0.410	0.254
WBS dup	chr7:72.7–74.3	2	0	NA	0.1007	0.044	0.016	0.016
16p11.2 dup	chr16:29.3–29.6	4	1	8.66	0.0377	0.059	0.000	0.087

^a^Results from previous reported European data sets (Br J Psychiatry. 2014;204(2):108–114). ^b^Frequencies of the CNVs in Chinese population, which were established from the Chinese study (Biol Psychiatry. 2016;80(4):331–337)

One out of 476 cases (0.21%) carried the 1q21.1 deletion, which was not expressed in any of the controls (*p* = 0.3175, Fisher’s exact test) (Table 1). The 1q21.1 deletion spans 1.3 Mb (chromosome 1: 146.5 Mb- 147.8 Mb) in the distal region of 1q21.1, not containing the TAR region. This deletion is also well known to be associated with SCZ, although the deletion region in our results indicated a much smaller area of deletion than Stephansson et al. described [6–8] The associated region is gene rich, and contains the gene BCL9, and when mutated, BCL9 has been shown by Li et al. to be associated with schizophrenia, as well as bipolar and major depression in the Chinese Han population [2]. However, the gene function for BCL9 is largely unknown. Besides schizophrenia, the highly unstable 1q21.1 region has also been implicated in a host of other human disorders, among which are idiopathic mental retardation [9, 10], autism/ASD [9], congenital heart disease [11], microcephaly/macrocephaly [9, 12] and neuroblastoma [13]. Dumas and Sikela indicated that the 1q21.1 CNV region contains the vast majority of DUF1220 protein domains, which portray a striking human lineage with a specific increase in copy number relative to the other great apes [14]. The increase in DUF1220 roughly correlates to increased brain size, and thus it is relevant to point out that deletions in 1q21.1 may also be associated with microcephaly, possibly due to a decrease in DUF1220. This trait is a common phenotype for schizophrenia as opposed to autism [15].

The 15q11.2 deletion from 22.7 Mb to 23.1 Mb occurred in one case (0.21%) and one control (0.098%) (*p* = 0.5344, Fisher’s exact test) (Table 1). The 15q11.2 deletion is one of the regions deleted in the Prader-Willi syndrome, whose symptoms include obsessive compulsive behavior, adaptive skills difficulty, and low intellectual ability. Of note, the Prader-Willi deletion in the Prader-Willi syndrome is much larger than that we identified. In addition, the 15q11.2 deletion has been reported in three different studies to be associated with SCZ [4]. The most relevant gene affected in the 15q11.2 region, CYFIP1 interacts with the fragile X mental retardation protein (FMRP) as well as the Rho GTPase Rac1, which is involved in the maintenance of neuronal structures [16].

We also observed that the Williams-Beuren syndrome (WBS) region from 22.7 Mb to 23.1 Mb on chromosome 7 was duplicated in two cases (0.42%), but not in controls (Table 1), for a p-value of 0.1007. The association between the 7q11.23 duplication and schizophrenia was previously reported by Mulle et al., [17] as well as Kirov et al [4]. Despite the fact that the specific biological pathways are unknown, the SCZ patients with WBS duplication also all had social anxiety and language delay prior to the onset of SCZ.

**Fig. 1:**
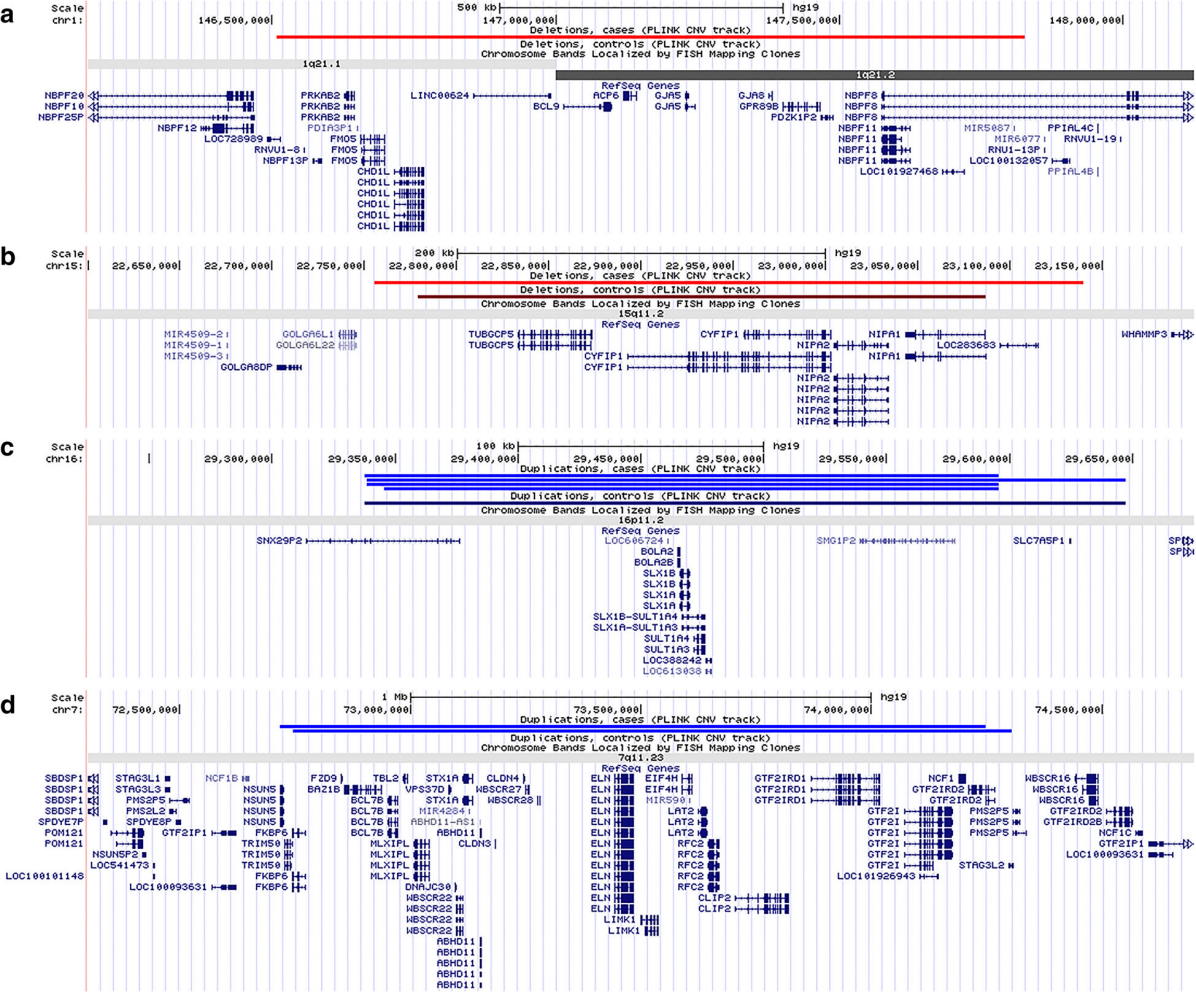
Schematic illustration of the chromosomal locations of the rare CNVs using UCSC Genome Browser. **a** 1q21.1 del; **b** 15q11.2 del; **c** 16p11.2 dup; **d** WBS dup. Note: In custom tracks, *red* color indicates deletion in case, *dark red* indicates deletion in control. For duplication *bright blue* color indicates duplication in case, while *dark blue* indicates duplication in control

Take all the four highlighted CNVs as a whole, 1.68% of our cases have a pathogenic CNV, it is about eight times higher than that in the control group (0.20%). The detailed locations are given in Fig. 1 and the Additional file 1: Table S2.

## Conclusions

To summarize, this study reported four CNV findings, including two deletions and two duplications. The results highlight the potential role of these deletions and duplications in the development of SCZ. Clearly, larger sample sized studies are needed for a careful localization of these CNVs and to possibly detect more deletions and/or duplications, associated with the development of SCZ in the Han Chinese population.

## Methods

We used a case/control paradigm, including 476 cases and 1023 controls (Additional file 1: Table S1). Before study enrollment, all SCZ patients and healthy subjects were required to sign a consent form. Diagnosis of SCZ was confirmed by interviews with at least two experienced psychiatrists in accordance with criteria in the *Diagnostic and Statistical Manual of Mental Disorders*, Fourth edition (DSM-IV). Exclusion criteria included the presence of other mood or neurodevelopmental disorders, epilepsy, or mental retardation. Control subjects were recruited by advertisement and selected based on the results from a questionnaire, concerning history of obstetric complications, substantial head injury, seizures, neurological or psychiatric disease, hypertension, diabetes, and substance abuse, as well as family medical histories. Subjects with any personal or family history of psychiatric illness among their first-degree relatives were excluded. The patients were recruited from several hospitals in Jiangsu province. All patients and healthy controls were age-matched and recruited from the cities of Wuxi and Nanjing in the Jiangsu province. Individuals not born in Jiangsu or whose family members were not born in Jiangsu were excluded from the study based on self-reported data. In addition, there was no blood relation between patients. All participants are Han Chinese. All samples were genotyped using the Axiom® Exome Genotyping Arrays. We first calculated the quality control measures (Dish-QC, DQC), and arrays with DQC < 0.82 (*n* = 26) signified poor quality, and could result in false-positive CNVs, so they were excluded from the study. We then called CNV for the left samples using PennCNV [18]. The genotyping calls and signal intensity data were generated using Affymetrix Power Tools, and then converts into Log R Ratio (LRR) and B Allele Frequency (BAF) values. To minimize the noise of genome-wide intensity signals, only samples within the s.d. of the normalized intensity (LRR < 0.35) were included. Wave artifacts roughly correlating with GC content resulting from hybridization bias of low full-length DNA quantity are known to interfere with the accurate inference of CNVs. Only samples where |GC base pair wave factor (GCWF)| < 0.05 were accepted. PLINK's identity-by-descent (IBD) analysis was used to detect cryptic relatedness based on the common variants (minor allele frequency > 1%) [19]. When a pair of individuals exhibited a PI_HAT > 0.2, the member of the pair with the lower call rate was excluded from the analysis (*n* = 3). The clean_cnv.pl program was adopted to merge adjacent calls of the same individual using the fraction threshold (0.5, calculated as base pair length). CNVs were then excluded if they were covered by < 5 probes or had a probe density > 1 probe/40 kb. If the count of CNV calls made by PennCNV exceeds 70, the DNA quality was assumed to be poor. Therefore, only samples with CNV call count <70 were included. After quality control, 476 cases and 1023 controls were kept for further analysis.

More than ten CNVs have been convincingly associated with SCZ [20, 21], and most of the previous studies are mainly carried out in Caucasian populations. We try to replicate the reported CNVs identified in Caucasian populations using a Han Chinese sample.

Four CNVs were found to be overrepresented in our cases and further evaluated as a result of our study, the 1q21.1 deletion, the 15q11.2 deletion, the WBS duplication and the 16p11.2 duplication. We compared the frequencies in the cases and controls groups for each particular region, and a Fisher's exact test was used to test the significance of the difference. All of these have been previously found to be associated with schizophrenia by multiple studies.

## Additional file

Additional file 1: Table S1. Characteristics of our sample. **Table S2.** The details for the highlighted CNVs in our sample. (DOCX 16 kb)

## Abbreviations

ASD: Autism spectrum disorder
CNV: Copy number variant
GWAS: Genome wide association study
SCZ: Schizophrenia
SNP: Single nucleotide polymorphism
WBS: Williams-Beuren syndrome
