# Supplementary material for: A replication study of schizophrenia-related rare copy number variations in a Han Southern Chinese population
Source: Hereditas. 2017 Jan 14;154:2. doi: 10.1186/s41065-016-0025-x (PMC5237532; doi:10.1186/s41065-016-0025-x)
Supplement: Additional file 1: Table S1. — Characteristics of our sample. Table S2. The details for the highlighted CNVs in our sample. (DOCX 16 kb) [file 41065_2016_25_MOESM1_ESM.docx]

**Table S1. Characteristics of our sample**

| **Group** | **N** | **Age** | **Female %** |
| --- | --- | --- | --- |
| Case | 476 | 45.1 ± 13.6 | 48.5 |
| Control | 1023 | 46.1 ± 12.0 | 48.7 |

**Table S2. The details for the highlighted CNVs in our sample**

| **GROUP** | **SAMPLE** | **CHR** | **BP1** | **BP2** | **Copy Number** | **Markers** |
| --- | --- | --- | --- | --- | --- | --- |
| Case | F09-wxs460_F09 | 1 | 146508774 | 147826275 | 1 | 319 |
| Case | E10-wxs158_E10 | 7 | 72717439 | 74247930 | 3 | 190 |
| Case | A06-wxs394_A06 | 7 | 72744236 | 74303860 | 3 | 174 |
| Case | B08-wxs409_B08 | 15 | 22755185 | 23140114 | 1 | 198 |
| Control | control3250 | 15 | 22755185 | 23086920 | 3 | 193 |
| Case | H02-wxs186_H02 | 16 | 29337658 | 29595483 | 4 | 10 |
| Control | control0305 | 16 | 29337658 | 29647340 | 3 | 13 |
| Case | G12-wxs376_G12 | 16 | 29338527 | 29595483 | 3 | 9 |
| Case | H03-wxs088_H03 | 16 | 29338527 | 29647342 | 3 | 12 |
| Case | H04-wxs089_H04 | 16 | 29345440 | 29595483 | 4 | 7 |
